# Effect of Geometry on Local Microstructure in Ti-6Al-4V Fabricated by Laser Powder Bed Fusion

**DOI:** 10.3390/ma18163756

**Published:** 2025-08-11

**Authors:** Chengshang Zhou, Noah Garcia, Runlin Pu, Pei Sun, Zhigang Zak Fang

**Affiliations:** Department of Materials Science and Engineering, The University of Utah, Salt Lake City, UT 84112-0114, USA

**Keywords:** additive manufacturing, laser powder bed fusion, Ti-6Al-4V, geometry

## Abstract

Laser powder bed fusion (L-PBF) is a unique technology that enables manufacturing geometrically complex metal alloys, including Ti-6Al-4V parts. The microstructure of Ti-6Al-4V is determined by its localized thermal history, which is affected by not only the L-PBF process but also the geometry of the part. Understanding the microstructure at specific locations in complex geometries is of great importance in predicting the mechanical performance of Ti-6Al-4V parts. This work investigates the effects of geometric features on the local microstructure. Three geometries, namely, holes, overhangs, and penholders, were designed and used for this study. Three different laser powers, namely 150 W, 250 W, and 350 W, were set to print those geometries. The use of a lower laser power results in improved print quality. While the martensite phase dominates the bulk of the L-PBF Ti-6Al-4V parts, a fine α+β lamellar structure can form at down-skin regions of printed horizontal holes and overhangs. Moreover, the direction of the columnar prime β grain can shift due to directional heat dissipation. The local microstructural evolution after heat treatment is investigated as well.

## 1. Introduction

Laser powder bed fusion (L-PBF) is an additive manufacturing technology that has gained significant attention in the last two decades due to its ability to produce high-quality and complex-shaped parts with a fast lead time [1,2,3,4]. Among various additively manufactured alloys, titanium alloys hold a special place due to the combination of their properties and cost, such as their outstanding strength-to-weight ratio, corrosion resistance, and biocompatibility, making them a top choice for critical components for a wide range of aerospace, medical, and automotive applications [5,6,7,8].

L-PBF selectively melts and builds parts layer-by-layer using a high-powered laser. This layer-by-layer approach allows one to fabricate intricate and custom-designed parts that would be challenging or impossible to produce using traditional manufacturing methods. However, the printability and microstructure control of titanium alloy parts with highly complex geometries have become two of the major concerns [9,10,11]. Fabrication of a complex geometry often induces more difficulties in controlling the microstructure, thermal distortion, and residual stresses during the L-PBF process [12]. When the stacking layers change or shift dramatically, in-process failures and defects, including distortion, cracking, warping, sagging, and delamination, can take place [13,14,15,16]. Moreover, Ti alloys can be more challenging due to their relatively low thermal conductivity [17]. As a result, the design of an L-PBF-manufactured Ti-6Al-4V part can be difficult. For example, designs with overhang features without support are generally limited to angles less than 45° [18,19]. Printed holes with their axial direction perpendicular to the build direction should be limited to <10 mm in diameter if there is no support [20,21].

Recently, innovative L-PBF strategies have emerged that can improve the printability of complex geometries. EOS has released “Smart Fusion” build control software to manage heat with a closed loop in real time, enabling one to not only reduce reliance on support construction but also print more challenging geometries [22]. Another strategy first introduced by EOS is called the “penholder” method, which allows for the printing of supportless parts with a high aspect ratio. The “penholder” method is of great interest because it does not use support structures to connect a part to the build plate, making platform removal easy [23]. Additionally, another commercial L-PBF machine manufacturer, SLM Solutions, independently developed a “Free Float” technology that enables a drastic reduction in the use of support structures and the printing of more complex geometries by optimizing scan strategies [24].

Despite the strategies mentioned above, the effects of the geometries on the microstructure of L-PBF Ti alloys remain less than predictable. It is logical to recognize that the thermal gradient during solidification in the L-PBF process leads to microstructural anisotropy, specifically the columnar prime-β grains in the Ti-6Al-4V alloy align with the build direction [25,26,27]. This microstructure anisotropy would consequently cause anisotropic mechanical properties. In addition, microstructural heterogeneity also exists due to the significant impact of geometry on localized thermal history [28,29,30]. In general, rapid cooling from temperatures above the martensitic start temperature (M_s_) leads to the formation of metastable α′ martensite in Ti-6Al-4V alloys [31]. A martensitic microstructure often results in high strength but brittle behavior [32]. Through suitable heat treatments, the martensite phase decomposes into an equilibrium α+β lamellar phase with desirable mechanical properties [33]. However, different microstructures may be obtained due to the layer-by-layer building process and the vertical thermal gradient. Recent research has revealed that an ultra-fine lamellar α+β structure can be obtained through in situ decomposition of the α′ martensite phase induced by the heating effect from the subsequent layer scans [34,35]. Such a heating effect has been recognized as an intrinsic heat treatment (IHT). It is believed that the fine lamellar α+β structure results in a good combination of mechanical properties.

Given the complexity of the L-PBF process, the effects of geometry on the local microstructure and the variations in microstructure in a printed part have been recognized in some reported studies [2]. Antonysamy et al. [36] found that in different regions of additively manufactured Ti-6Al-4V parts, there are two types of distinct microstructure, namely the coarse columnar β grain form and fine equiaxed β grain form. A curved fiber-like texture appeared in the bulk, down-skin, and side-contour sections. Xu et al. [35] observed that equiaxed β grains are present in thin as-built Ti-6Al-4V samples. Also, there is the effect of a part’s dimensions on the microstructure, i.e., α′ martensite is retained in smaller cylindrical rods and a fine lamellar α+β structure is formed in larger rods. Recently, Barriobero-Vila et al. [14] investigated the α′ decomposition and localized spheroidization of a Ti-6Al-4V impeller fabricated by L-PBF. It was observed that martensite decomposition took place in the central region, showing a lamellar α+β structure, while the up-skin region consisted of an α′ phase due to there being no heat accumulation. The down-skin region showed a higher volume fraction of β grains and tended to coarsen and spheroidize.

Although the effects of geometry on the microstructure of L-PBF Ti-6Al-4V are well recognized, predicting such effects and the final microstructure remains challenging. There is a lack of systematic studies that could correlate the geometric features to the microstructure. Here we present investigations regarding the effect of geometry on the local microstructure of L-PBF Ti-6Al-4V. We use three types of geometries—hole, overhang, and penholder—that represent a broad range of geometric features to shed light on the corresponding microstructures. First, the printability and fidelity of the designed geometries are examined. Second, microstructural features such as the columnar β grains, α′ martensite, IHT-induced fine lamellar α+β structure, and heat-treated lamellar α+β structure are identified and correlated to geometric features. Our work aims to provide a better understanding of the relation between laser parameters, geometry, and the local Ti-6Al-4V.

## 2. Experimental Section

### 2.1. Fabrication

Laser powder bed fusion was performed on an SLM-125HL machine (SLM Solutions Group AG, Lübeck, Germany) equipped with a maximum laser power of 400 W. Prior to the L-PBF process, a high-purity argon gas was used to flood the system to reduce the oxygen level to below 0.02 vol.% in the build chamber. During L-PBF, a titanium substrate plate was employed and preheated to 200 °C to mitigate the residual stress during printing.

Ti-6Al-4V alloy powder with a specific size distribution (D_10_ = 24 μm, D_50_ = 35 μm, D_90_ = 48 μm) was purchased from AP&C Inc. (St-Eustache, QC, Canada). The oxygen content of the powder was 0.12 wt%. Our preliminary study determined the process window for printing Ti-6Al-4V alloys. According to the process window, three types of scan parameters were used, as summarized in Table 1. The difference between the three strategies is that the laser powers of the volume scan are set to be 150 W, 250 W, and 350 W.

### 2.2. Geometries

**Figure 1:**
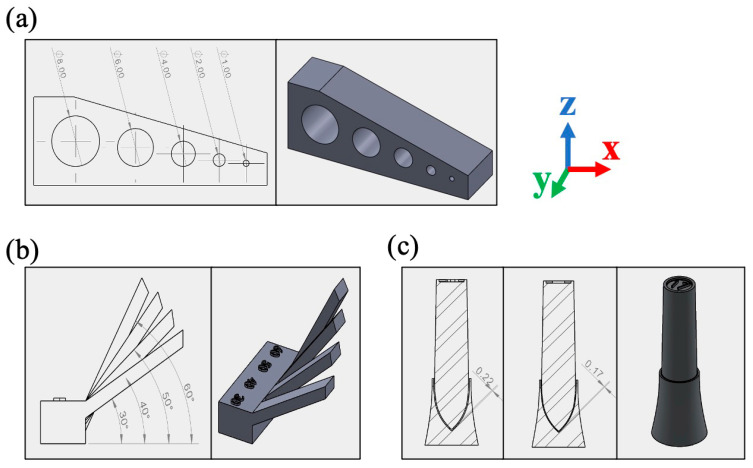
Designed geometries of (**a**) a block with horizontal holes of 1 mm, 2 mm, 4 mm, 6 mm, and 8 mm diameters; (**b**) overhangs with angles of 30°, 40°, 50°, and 60°; (**c**) penholder parts with gaps of 0.17 and 0.22 mm.

Three sets of geometries, namely, overhangs, holes, and penholders, were designed and fabricated using the SLM-125 machine. As shown in Figure 1, the hole design includes Φ1 mm, Φ2 mm, Φ4 mm, Φ6 mm, and Φ8 mm printed horizontal holes. The overhangs consist of four different angles, which are 30°, 40°, 50°, and 60°. The penholder design consists of a pen-like rod and a holder part, where the gap between the pen and holder is set to be 0.17 mm or 0.22 mm. All geometries are denoted in Table 2, along with their respective features and parameters. The term “up-skin” refers to the area where the normal direction of the part’s surface forms an acute angle with the positive direction of the structure’s *z*-axis. The term “down-skin” refers to the area where the normal direction of the part’s surface forms an acute angle with the negative direction of the structure’s *z*-axis.

### 2.3. Characterization

Density measurements were obtained using Pycnometer (Micrometrics, AccuPyc II 1340, Norcross, GA, USA). The as-built and heat-treated specimens underwent cutting, mounting, polishing, and etching using Kroll solution for metallographic examination. The optic microscopy (OM) analysis was carried out using Keyence (Keyence, VHX-5000, Kanagawa, Japan). The SEM imaging was carried out by using an SEM (FEI Nova Nano 630, Hillsboro, OR, USA).

## 3. Results

### 3.1. Print Quality

Before we present the effects of geometry on the microstructure of L-PBF Ti64, we examine the macro effects of geometry on the quality of the prints under different laser powers and scan speeds. As mentioned in the experimental section, we chose three different geometric designs for this study: internal holes perpendicular to the build direction, overhangs, and penholder designs. Internal holes and overhangs are common geometric features in part designs, and the penholder design is a technique used to minimize the use of support structures in L-PBF.

The samples (H-150, H-250, and H-350) with internal holes (Φ1, 2, 4, 6, 8 mm) were printed perpendicular to the build direction. Three laser parameters, namely, 150 W, 250 W, and 350 W, were used in this study. The densities of the printed H-150, H-250, and H-350 samples are 4.40 g/cm^3^ (99.3%), 4.39 g/cm^3^ (99.1%), and 4.38 g/cm^3^ (98.9%), respectively.

**Figure 2:**
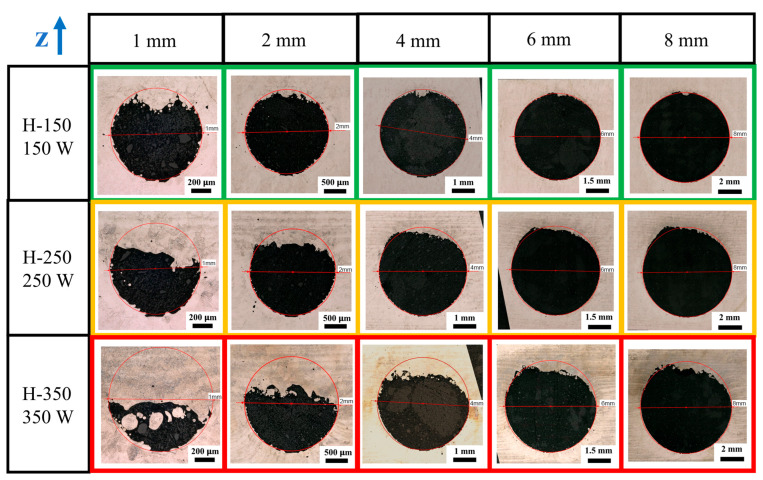
Optic microscopy photos of Φ1 mm, 2 mm, 4 mm, 6 mm, and 8 mm horizontal holes with laser power of 150 W, 250 W, and 350 W.

Figure 2 shows cross-sectional views of the holes. First, it can clearly be seen that the down-skin regions of all the holes are rougher than the side-skin and up-skin regions, demonstrating the challenge of printing holes with the axial direction of the holes perpendicular to the build direction without adding a support structure. The use of a lower laser power (150 W) resulted in better shape fidelity than those printed via higher laser powers. Moreover, the holes printed using 250 W and 350 W show “sagging” down-skin regions. This is because a high laser power generates an excessive melting pool, which tends to sink. For the 1 mm diameter hole in the sample H-350, over half of the hole was occupied by the sagging material, resulting in poor shape fidelity. For the holes in the H-350 sample, the thickness of the sagging mass was in the range of 0.5–1 mm. For the holes in H-250, the thickness of the sagging mass was in the range of 0.3–0.5 mm. The change in the hole diameter seems to have no impact on reducing the sag, but the larger holes appear to have better shape fidelity due to the size increase.

**Figure 3:**
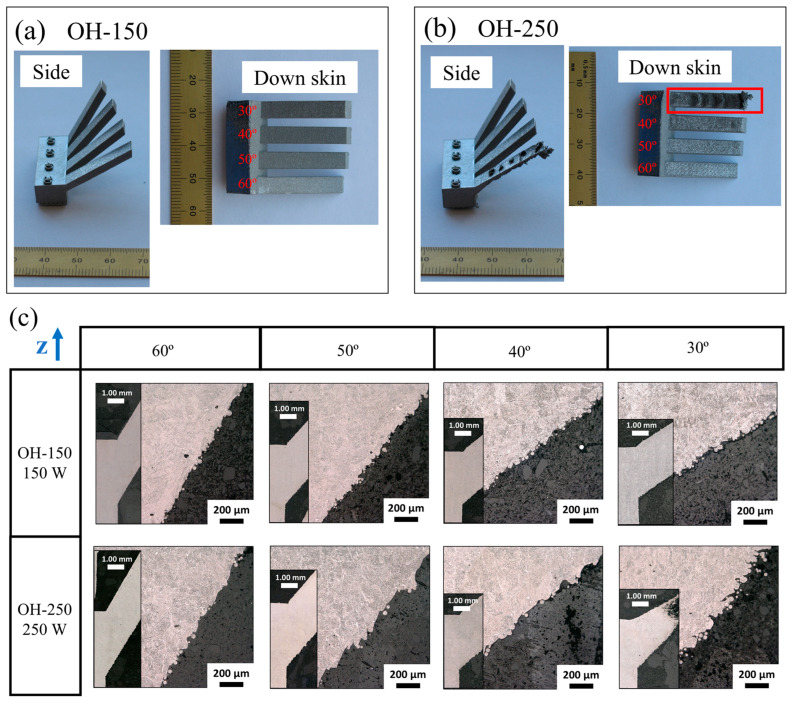
Overhangs that were printed using 150 W (**a**) and 250 W (**b**) laser power and (**c**) the OM images of the down-skin section.

We printed the overhang samples with four different angles (30°, 40°, 50°, and 60°). And two overhang parts were printed using 150 W (OH-150) and 250 W (OH-250) laser powers, as shown in Figure 3a. The sample using 350 W experienced an in-process failure. The OH-250 sample with a 30° overhang suffered a failure as well, due to a bad down-skin surface and a collapsed area (the red box in Figure 3b). Except for the 30° overhang in the OH-250 sample, all other overhang geometries were printed successfully. Figure 3b provides cross-sectional views of the down-skin areas for each overhang. It shows that the overhang samples with higher angles (60°) had better surface finishes for the down-skin areas. The roughness of the down-skin area is mainly attributed to the partially fused particles adhering to the surface. Compared to the down-skin area of the OH-250 samples with a 250 W laser power, the down-skin areas of the lower-laser-power printed parts (OH-150) exhibited better surface quality.

**Figure 4:**
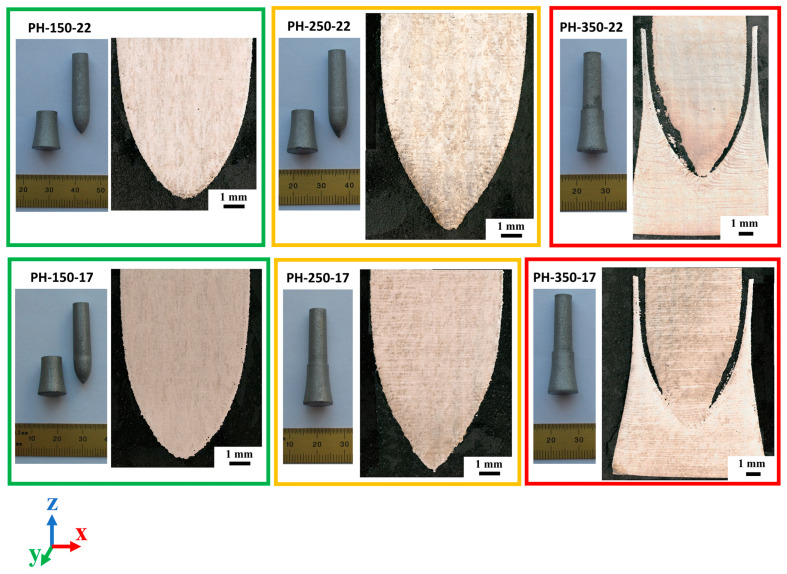
Penholders that were printed using 150 W, 250 W, and 350 W laser power and the OM images of the building section.

The purpose of the “penholder” strategy is to design a support-free L-PBF part that can be readily detached after the printing process. We designed two “penholders” with 0.17 mm and 0.22 mm gaps. The penholder samples were printed using 150 W, 250 W, and 350 W laser powers, and section images are shown in Figure 4. After printing, the pen bodies of the PH-150-17, PH-150-22, and PH-250-22 samples could easily be detached from the holders. The pen body of the PH-250-17 sample could also be detached using tools. However, the sample printed using a higher laser power, 350 W, did not detach easily, indicating that the pen bodies and holders are partially fused. The OM images of the PH-350-17 and PH-350-22 samples confirm that there are fused regions between the pen body and holder. Figure 4 also shows the very rough down-skin regions and asymmetrical shapes of the PH-350-17 and PH-350-22 pen bodies. Obviously, the laser power and laser energy density are critical factors that determine the quality of the “penholder” print. Also, it should be noted that there is a small window for the gap size when using the “penholder” strategy. A narrow gap design would easily lead to fusion, whereas a wide gap design may not provide a successful holding effect for the “pen” geometry. A successful “penholder” strategy must balance the laser energy density applied to the down-skin area and the designed gap, which affect the heat dissipation. This may lead to a possible optimized strategy for the “penholder” design. For example, reduced laser energy for the down-skin scans and the adjacent volume can be utilized to improve the quality of the “penholder” prints.

### 3.2. Effects of Geometry on the Microstructure

#### 3.2.1. Prime-β Grains

**Figure 5:**
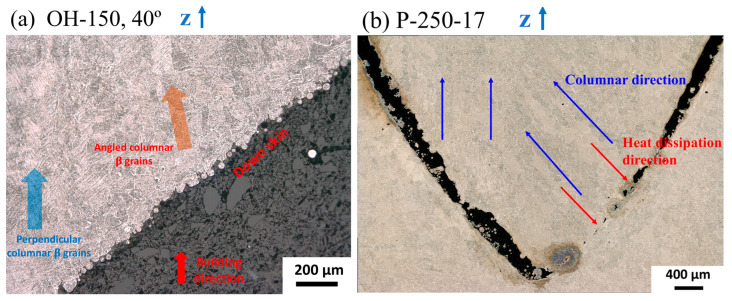
Columnar prime-β grains affected by geometries: (**a**) the 40° overhang OH-150 and (**b**) the partially fused penholder P-250-17.

Columnar prime-β grains along the building direction are one of the most important microstructural features in an L-PBF Ti64 alloy. The formation of the columnar grains is associated with the steep thermal gradient generated during deposition. Based on our OM and SEM characterization, the columnar prime-β grain structure is observed in all samples. The columnar grain texture is found to be parallel to the building direction and is perpendicular (90°) to the deposition plane. However, we found that the direction of a columnar grain structure may shift in specific regions of a part due to the change in the direction of thermal dissipation, which is related to the geometry. Two examples are provided in Figure 5. First, Figure 5a presents the near down-skin region of the 50° overhang in the OH-150 sample, showing that the direction of the columnar grain inclines at an angle against the overhang. The incline angle is estimated to be 10–15°. Second, the direction of the columnar grain can shift significantly in the region where two parts are fused together, as exemplified by the penholder parts. Figure 5b shows the partially fused area in the PH-250-17 samples. The pen body and holder partially fused together, leading to a directional shift in the columnar grains above the fused part. This should be attributed to the direction of heat dissipation shifting due to geometry. In both cases, the height of affected regions is in the range of 1000–2000 μm. It is worth noting that such a microstructure will have an impact on the mechanical performance of the parts, especially for parts that will serve their purpose without surface finishing.

**Figure 6:**
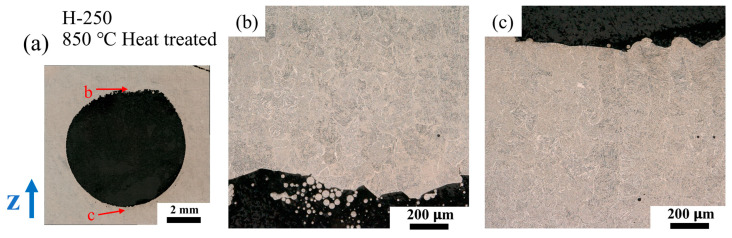
OM image of 850 °C heat-treated H-250 sample (**a**): down-skin area (**b**) and up-skin area (**c**).

Further examination shows that equiaxed grains can also be found in some specific regions, e.g., the 100–200 μm layer in the down-skin area of the 30° overhang in the sample OH-350, and the 100–300 μm region at the bottom of the part adjacent to the support structure. Equiaxed prime-β grains were also found in the down-skin region of the holes. Figure 6 compares the prime-β morphology between the down-skin and up-skin regions. The H-250 sample was heat treated at 850 °C for 2 h so that the prime-β grains could be clearly observed in the OM images. It is obvious that near the down-skin region, the prime-β grains are equiaxed and in a size range of 100–200 μm, while the up-skin region consists of columnar prime-β grains.

#### 3.2.2. Martensitic and Intrinsic Heat-Treated (IHT) Microstructure

**Figure 7:**
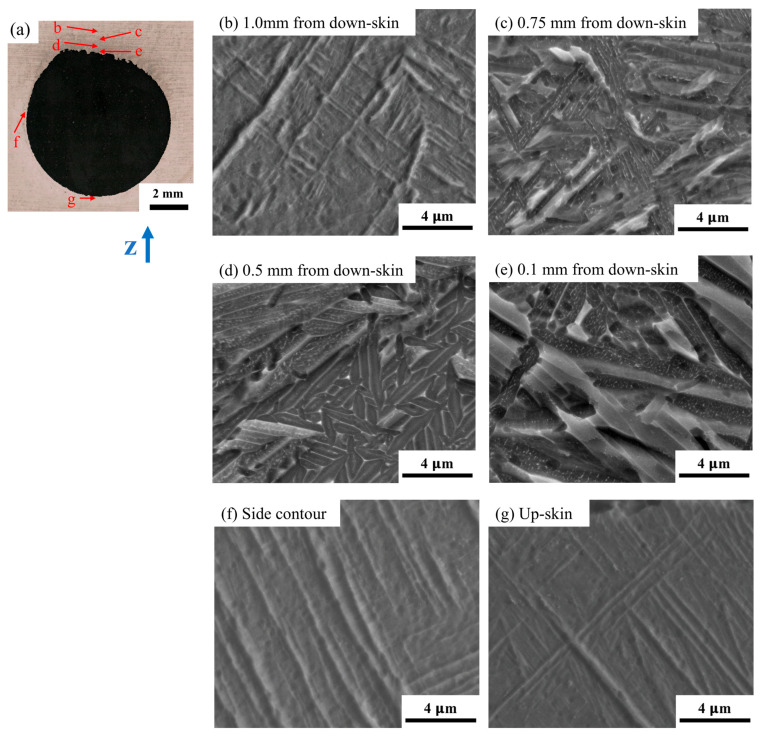
Local microstructure around the Φ8 mm hole on the H-250 sample. (**a**) The OM image of the hole; the SEM images at (**b**) 1.0 mm from the down-skin region (up the contour of the hole), (**c**) 0.75 mm from the down-skin region, (**d**) 0.5 mm from the down-skin region, (**e**) 0.1 mm from the down-skin region, (**f**) the side-contour region, and (**g**) the up-skin region.

Due to the inherent high cooling rate, acicular or plate martensite (α′) dominates in the microstructure of the L-PBF Ti-6Al-4V alloy. Recently, a phenomenon called intrinsic heat treatment (-) or in situ α′ martensite decomposition was widely observed and reported. IHT usually results in a refined α+β lamellar structure and can be obtained by controlling the L-PBF processing variables [34,35]. This study examines the local microstructure features, including the martensite and fine lamellar α+β structure, as affected by the designed geometric features. Figure 7 presents high-resolution SEM microstructure images corresponding to different locations in the H-250 sample, i.e., the 8 mm hole sample. First, the microstructure variations as a function of the distance from the down-skin surface up to 1 mm are shown in Figure 7b–e. In the area close to the down-skin region (0.1 mm above, Figure 7e), we observed a typical lamellar α+β microstructure, with an α-lath width of 0.4–0.8 μm. This indicates a significant IHT effect due to poor heat dissipation in the “roof” area of the hole geometry. In the area 0.5 mm away from the down-skin region (Figure 7d), a lamellar α+β structure was observed, but it was finer than the microstructure shown in Figure 7e. Additionally, it is also noted that a fine Widmanstatten α+β structure (α-lath width < 0.5 μm) partially forms in that area. At a distance of 0.75 mm to the down-skin region (Figure 7c), a mixture of α′ martensite and decomposed α+β phases was identified. The α+β structure is very fine and unique because it seems to be at a very early stage of α′ martensite decomposition, featuring a 100–300 nm α-lath width and some discontinuous β phase. The fine lamellar α+β structure is the result of the cyclic heating effect caused by sequential laser scans. The lamellar α+β microstructure evolves at different stages during IHT. Further away from the down-skin region at a 1.0 mm distance (Figure 7b), we found a full α′ martensite structure. However, we found that the α′ martensite structure in Figure 7b is different from the α′ martensite structure in the side-contour (Figure 7f) and up-skin sections (Figure 7g). The former consists of shorter, finer, and basketweave-like α′ martensite, while the latter consists of much longer and coarser α′ needles. The microstructural difference above should be attributed to the different solidification and IHT conditions at different locations.

In the up-skin and side-contour regions, the solidification cooling rate was relatively faster, so the IHT effect was minimal compared to the region adjacent to the down-skin area. On the contrary, a slower cooling rate occurred in the down-skin area; thus the IHT effect was enhanced. It is suggested that for one complex geometry, different areas may have significant differences in terms of their localized thermal history, which governs the local microstructure and mechanical performance.

**Figure 8:**
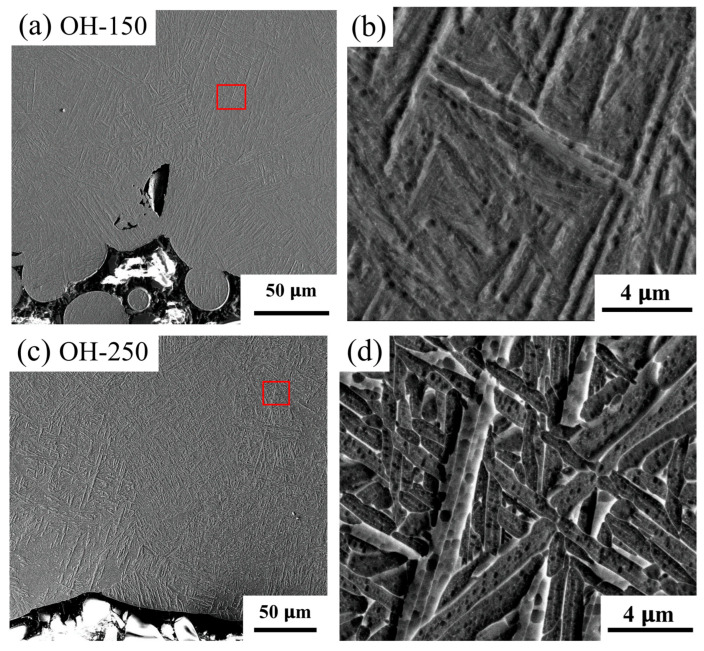
Microstructure adjacent to the 30° angle down-skin region of the OH-150 (**a**,**b**) and OH-250 (**c**,**d**) overhangs. The red squares in (**a**,**c**) indicate the locations where the (**b**,**d**) images were taken.

Further examination indicates that the holes with different diameters (1–8 mm) all have a lamellar α+β microstructure; however, the smaller holes present slightly smaller lamellar α+β areas. However, we observed that the lamellar α+β regions were significantly reduced when a lower laser power was applied (H-150). On the contrary, the lamellar α+β regions were increased significantly when a higher laser power was used (350 W, H-350). Moreover, the lamellar α+β structure can be found in the down-skin region in the overhang part OH-250. Figure 8 compares the microstructures near the down-skin regions of the 30° overhangs (OH-150 and OH-250). A lamellar α+β layer with a 200–300 μm thickness was found in the down-skin regions of the 30 and 40° overhangs in the OH-250 sample. For the OH-150 part, we found that the down-skin area mainly consists of α′ martensite and no α+β structure, indicating a very limited IHT effect due to the use of a lower laser power.

### 3.3. Effect of Heat Treatment

**Figure 9:**
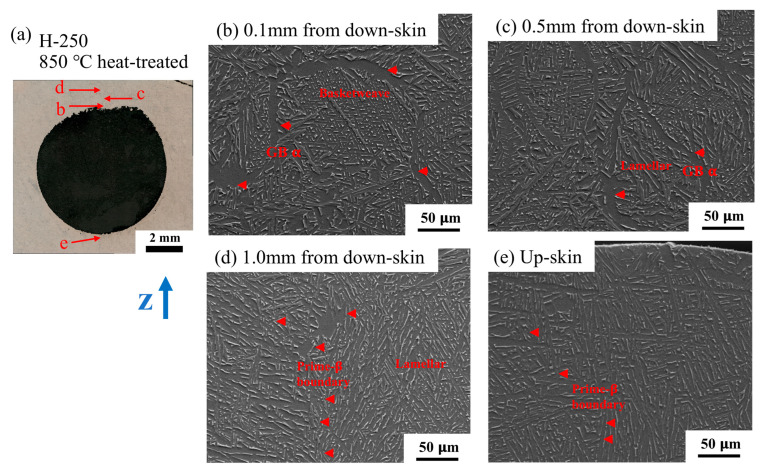
Local microstructure of the H-250 sample after heat treatment at 850 °C: (**a**) section image of the 8 mm hole, (**b**) 0.1 mm above the down-skin surface, (**c**) 0.5 mm above the down-skin surface, (**d**) 1 mm above the down-skin surface, and (**e**) the up-skin region.

The H-250 sample was heat treated at 850 °C for 2 h to investigate the effect of heat treatment on different geometric microstructures [33]. Figure 9 presents the local heat-treated microstructure that is comparable to the as-built microstructure (Figure 7). One of the interesting findings is the dramatic growth of the grain-boundary α (GB-α) phase in the region of the down-skin area, as shown in Figure 9b,c. The GB-α phase is continuously formed at the prime-β boundary with over a 10 μm thickness. In contrast, the up-skin region (Figure 9e) and the region 1 mm from the down-skin area (Figure 9d), which previously consisted of an α′ martensite phase in the as-built microstructure, undergo much less or even no GB-α formation during heat treatment. It seems that the IHT-induced α+β lamellar structure promoted GB-α growth during the 850 °C heat treatment. It has been reported that the GB-α phase, especially during the formation of the continuous GB-α layer, has a deleterious effect on the mechanical properties, including lowering ductility, lowering fracture toughness, and reducing fatigue resistance [37,38,39]. Prithiv et al. [37] suggested that the formation of the GB-α phase could be related to some α-stabilizing elements at the boundaries, especially oxygen. A higher oxygen concentration in the down-skin region is expected because of the oxygen impurity in the argon atmosphere. Discoloration was often observed on the down-skin surface. However, a more detailed analysis is necessary to determine the exact oxygen content in the down-skin regions. In addition, the α+β structure is also different in various regions, as shown in Figure 9b–e. We found fewer α colonies but a large amount of basketweave-like α+β colonies in the region close to the down-skin area (Figure 9b). In contrast, the up-skin region (Figure 9e) and regions far away from the down-skin area (Figure 9c,d) show predominantly α+β lamellar structures.

## 4. Discussion

The microstructure of L-PBF Ti-6Al-4V alloys depends on their thermal history. Therefore, process parameters, such as laser power and scan speed, should determine the bulk microstructure. However, when the process parameters are fixed, geometric features may result in different thermal histories in localized regions, thus causing different microstructures. Table 3 summarizes the shape fidelity problems and corresponding microstructural features of different geometries. Munk et al. [28] demonstrated a similar trend regarding microstructural differences, namely an α+β phase structure at locations with a higher thermal input compared to an α′ phase structure at locations with a common thermal input. Their observation also showed that a significant microstructural difference can be found even at a distance of 5 mm in the building direction. In this study, we found a general trend that the α+β lamellar structure tends to appear in regions with poor heat dissipation, which are generally the down-skin regions of the holes and overhangs. The excessive heat from subsequent scans caused a significant IHT effect, and this feature also leads to a difference in the heat-treated microstructure.

**Figure 10:**
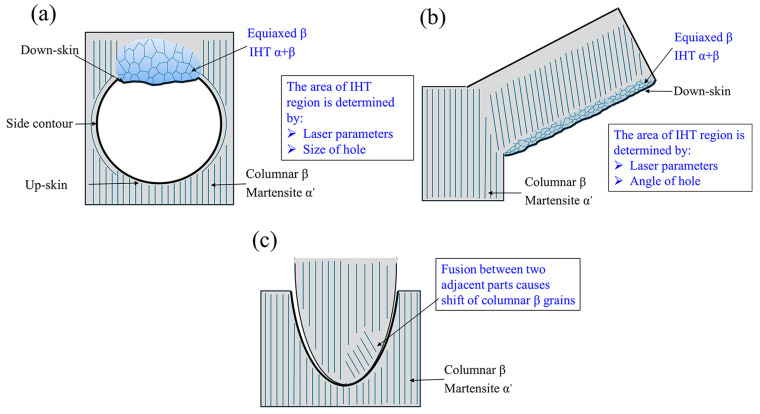
Schematics show the features of the local microstructure for (**a**) printed horizontal hole, (**b**) overhang, and (**c**) penholder parts.

Moreover, we found that the columnar grain structure also responded to the geometries, which were also associated with heat dissipation during L-PBF. Figure 10 provides schematics showing a general trend, namely that certain microstructures can be formed at different locations with different geometries. Note that we did not identify a lamellar α+β microstructure in the down-skin region of the penholder structure, suggesting that the cooling rate in that area was relatively good.

The microstructure of the alloy determines the mechanical behavior of Ti-6Al-4V parts. In most cases, additively manufactured parts with a complex geometry will not undergo machining, so the as-printed surface microstructure will significantly affect performance. A fully martensitic microstructure is usually recognized as hard and brittle with high strength and low ductility, although there is recently reported research showing that the α′ martensitic structure may not be inherently brittle and could have good ductility (~10% elongation) [40,41]. Table 4 summarizes the reported mechanical properties corresponding to martensitic, fine α+β lamellar, and coarse α+β lamellar microstructures. The IHT effect produces a fine lamellar α+β structure, featuring a good combination of strength and ductility [34,35]. Furthermore, heat treating the L-PBF Ti-6Al-4V alloy above the martensite decomposition temperature (600–850 °C) generally produces an α+β lamellar structure. Higher annealing temperature results in a coarser lamellar structure associated with decreased strength and improved ductility [32].

## 5. Summaries

In summary, we investigated the effect of geometry on local microstructures for three types of geometry features, including holes, overhangs, and penholders. The following results were observed.

(1) While columnar prime-β grains form in the bulk and up-skin regions, equiaxed prime-β grains can be found in specific down-skin regions lacking support. The equiaxed region is linked to local heat dissipation during L-PBF.
(2) The direction of columnar prime-β grains can shift at specific locations, such as the down-skin region of overhangs and the partially fused region in penholders.
(3) The IHT effect was identified in specific down-skin regions. We observed a gradual transition from a martensite decomposed α+β lamellar structure to a fully α′ martensite structure in the down-skin region of horizontal holes.
(4) A thick grain-boundary α phase (GB-α) layer tended to form in the down-skin regions after heat treatment at 850 °C.

## Figures and Tables

**Table 1:** The processing parameters used in the L-PBF process.

	P, Laser Power (W)	V, Scanning Velocity (mm/s)	H, Hatch Distance (mm)	T, Layer Thickness (μm)	VED, Volumetric Energy Density (J/mm^3^)	FOD, Focal Offset Distance (mm)
Volume scan	150	1000	0.12	30	41.67	0
	250	1000	0.12	30	69.44	0
	350	1000	0.12	30	97.22	0
Contour scan	80	450	0.12	30	49.39	0
Up-skin scan	100	465	0.12	30	59.74	0
Down-skin scan	75	1000	0.12	30	20.84	−8

**Table 2:** Summary of geometry features and the laser parameters for each sample.

Parts	Laser Power, W	Designation	Geometry Description
Horizontal holes	150	H-150	Hole diameter: 1 mm; 2 mm; 4 mm; 6 mm; 8 mm
	250	H-250	
	350	H-350	
Overhangs	150	OH-150	Angle: 60°; 50°; 40°; 30°
	250	OH-250	
Penholders	150	PH-150-17	Gap: 0.17 mm
		PH-150-22	Gap: 0.22 mm
	250	PH-250-17	Gap: 0.17 mm
		PH-250-22	Gap: 0.22 mm
	350	PH-350-17	Gap: 0.17 mm
		PH-350-22	Gap: 0.22 mm

**Table 3:** Summary of microstructural features for different geometries.

Geometry	Shape Fidelity Problem	Microstructure Feature
Horizontal holes	“Sagging” down-skin surface on high-VED print	Equiaxed β grain Fine lamellar α+β structure in down-skin region
Overhangs	Poor down-skin surface on high-VED print	Equiaxed β Fine lamellar α+β structure in down-skin region Inclined β grain
Penholders	Fused parts on high-VED print	Inclined β grain

**Table 4:** Summary of typical mechanical properties corresponding to martensitic, fine α+β lamellar, and coarse α+β lamellar microstructures.

Process	Microstructure	Yield Strength, MPa	Ultimate Tensile Strength, MPa	Elongation, %	Ref.
L-PBF	Martensite α′	1195	1269	5.0	[42]
L-PBF	Fine lamellar α+β	1112	1165	11.6	[35]
L-PBF	Fine lamellar α+β	960	1058	14.1	[43]
L-PBF, heat treated at 700 °C	Martensite α′	1051	1115	11.3	[44]
L-PBF, heat treated at 800 °C	lamellar α+β	953	1050	14.7	[32]
L-PBF, heat treated at 900 °C	Lamellar α+β	899	1046	19.2	[45]
L-PBF, HIPed	Lamellar α+β	885	973	19.0	[44]

## Data Availability

The original contributions presented in this study are included in the article. Further inquiries can be directed to the corresponding author.
